# New View on the Initial Development Site and Radiographic Classification System of Osteoarthritis of the Knee Based on Radiographic Analysis

**Published:** 2012-12

**Authors:** Ki-Ho Moon

**Affiliations:** *Department of Orthopedic Surgery, Interfaith Medical Center, 1545 Atlantic Ave, Brooklyn, NY, USA*

**Keywords:** axis of the knee, initial development site of knee osteoarthritis, knee kinematics, narrowing of compartment and marginal osteophyte, osteoarthritis of the knee, osteophyte at tibial spine, radiographic classification system of knee osteoarthritis

## Abstract

**Introduction:**

Radiographic pathology of severe osteoarthritis of the knee (OAK) such as severe osteophyte at tibial spine (TS), compartment narrowing, marginal osteophyte, and subchondral sclerosis is well known. Kellgren-Lawrence grading system, which is widely used to diagnose OAK, describes narrowing-marginal osteophyte in 4-grades but uses osteophyte at TS only as evidence of OAK without detailed-grading. However, kinematically the knee employs medial TS as an axis while medial and lateral compartments carry the load, suggesting that early OAK would occur sooner at TS than at compartment. Then, Kellgren-Lawrence system may be inadequate to diagnose early-stage OAK manifested as a subtle osteophyte at TS without narrowing-marginal osteophyte. This undiagnosed-OAK will deteriorate becoming a contributing factor in an increasing incidence of OAK.

**Methods:**

This study developed a radiographic OAK-marker based on both osteophyte at TS and compartment narrowing-marginal osteophyte and graded as normal, mild, moderate, and severe. With this marker, both knee radiographs of 1,728 patients with knee pain were analyzed.

**Results:**

Among 611 early-stage mild OAK, 562 or 92% started at TS and 49 or 8% at compartment. It suggests the initial development site of OAK, helping develop new site-specific radiographic classification system of OAK accurately to diagnose all severity of OAK at early, intermediate, or late-stage. It showed that Kellgren-Lawrence system missed 92.0% of early-stage mild OAK from diagnosis.

**Conclusions:**

A subtle osteophyte at TS is the earliest radiographic sign of OAK. A new radiographic classification system of OAK was suggested for accurate diagnosis of all OAK in severity and at stage.

## INTRODUCTION

In 1957 Kellgren and Lawrence (K&L) introduced a grading system to diagnose osteoarthritis of hand, hip, wrist etc., based on narrowing-marginal osteophyte and for the knee, osteophyte at TS was mentioned as radiographic evidence of OAK (1). This K&L grading system was adapted at the World Health Organization meeting in 1961 where osteophyte at TS appears to be included in grade 1 and 2 but not in grade 3 and 4 (2). Thus, the description of osteophyte at TS in its use in K&L system was vague and general. Yet this system has been used in most study of OAK (3-5). Recently its inexact wording in description was raised urging a need of valid classification system (6, 7).

This vague description of osteophyte at TS may relate to scanty knowledge about TS. Recent edition of Gray’s anatomy (8) described TS only as “intercondylar eminence of the tibia” although Giorgi in 1956 reported its morphologic variation (9). Thus, TS has been treated as a mere bump on tibial plateau until 1975 when Girgis (10) described its anatomy and relationship with anterior cruciate ligament and anterior horns of menisci.

Pathologically OAK by definition is a progressive loss and attempted repair of articular cartilage (AC) and degeneration of meniscus (14, 15). In 1941 kinematically Brantigan & Voshell (11) established medial (M) TS as instantaneous centers of transverse axis of rotation of the tibia. Shaw *et al*. in 1974 (12) and Trent *et al*. in 1976 (13) reaffirmed MTS as knee axes. Thus, TS was established as an essential structure in knee kinematics more than 70 years ago. Then, according to Brantigan and others, it is TS through which tibia rotates and compartments bear the load at gait. Then, AC lined over TS would elicit osteophyte sooner than AC and meniscus of compartment elicits narrowing-marginal osteophyte just as hinges of a door get rusted and worn sooner than rims of a door. It shows that osteophyte at TS is earlier OAK pathology than narrowing-marginal osteophyte. Thus far, an impact of inexact description of osteophyte at TS in K&L system has been substantial: having kept focus only on compartment change as it is seen on the study of OAK on radiographs (3-5) or MRI (16) while having unnoticed and missed early mild OAK manifested as a subtle osteophyte at TS from diagnosis for more than half century. Since OAK is a progressive disease, this missed OAK will deteriorate. It may be one of reasons why the incidence of OAK is on a rise and demand for primary total knee arthroplasty is expected to grow fastest among patients who are 45-54 years old anticipating a 17-fold increase by 2030 (17).

This report offers a novel tool, called “radiographic OAK-marker” based on both osteophyte at TS and narrowing-marginal osteophyte at compartment developed from analyzing a series of well-documented advanced OAKs and normal knees. With this marker, 1,728 patients’ both knee radiographs were analyzed to define the initial OAK development site. Based on this, a new site-specific radiographic classification system of OAK was developed. It allows for accurate diagnosis of OAK of any severity at early, intermediate, or late stage eliminating a difficulty in diagnosis of OAK presented by K&L system.

## MATERIALS AND METHODS

To address the issues related to missed-OAK, a random non-biased cohort of 1,872 patients’ bilateral knee radiographs (anterior-posterior view with extended knee in supine) was obtained. Out of this set, well-documented cases of 84 severe OAK and 227 moderate OAK and 198 normal knees were analyzed to develop a novel radiographic OAK-marker.

### A Novel Radiographic OAK-Marker

**Figure 1:**
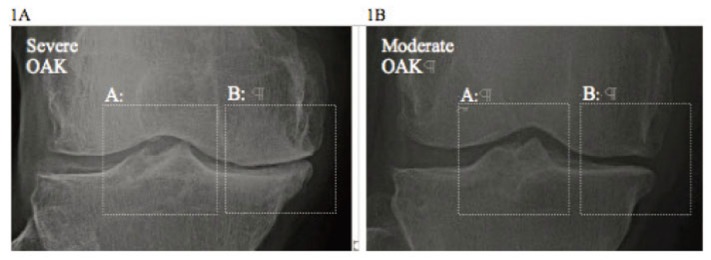
1A: the pathology of severe OAK shows: A) Osteophyte at TS; and B) narrowing and marginal osteophyte at compartment; 1B: the pathology of moderate OAK shows: A) Osteophyte at TS; and B) narrowing and marginal osteophyte at compartment.

A method how to develop a radiographic OAK-marker was the following: First, unlike hip, shoulder etc., kinematically the knee employs TS as an axis (13-15) while medial and lateral compartments carry the load at gait. Thus, radiographs of severe and moderate OAK selected were analyzed in kinematical aspect. It showed two pathological features in site (Fig. 1): A) osteophyte at TS; and B) unilateral narrowing-marginal osteophyte at compartment. It showed that the height of TS and narrowing-marginal osteophyte were more profound in severe OAK than in moderate one. If the height of osteophyte at TS and narrowing-marginal osteophyte at compartment is smaller than in moderate OAK, then, it should be graded as “mild” OAK.

**Figure 2:**
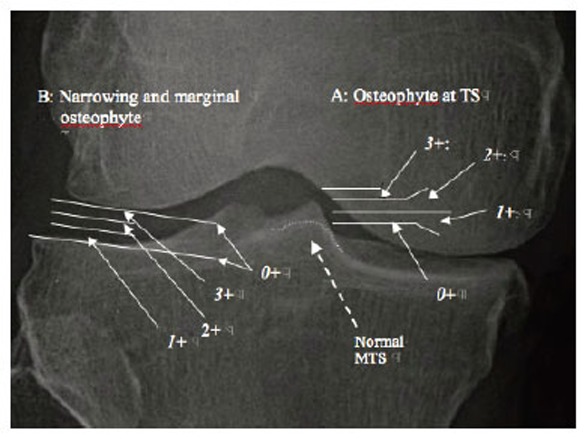
Radiographic OAK-marker in three grades: A: osteophyte at TS; and B: narrowing and marginal osteophyte at compartment. A dotted line at MTS indicates the borderline of normal MTS before developing osteophyte over it.

Objectively to define the severity of radiographic OAK pathology as mild, moderate, and severe, certain criteria were established as following (Fig. 2):

1. For osteophyte at TS (Fig. 2A), femoral intercondylar roof was used as a basis to assess the height of TS: no osteophyte at TS or *0*+ was defined as normal; when the apex of a taller MTS or LTS extended to less than 1/3 of a total distance between the apex and femoral intercondylar roof, it was marked *1*+ and graded as mild; when the apex of TS extended to more than 1/3 but less than 2/3, it was marked *2*+ and graded as moderate; and when the apex of TS extended more than 2/3, it was marked *3*+ and graded as severe.
2. For narrowing and marginal osteophyte at compartment (Fig. 2B), the space of non-narrowed contralateral compartment of same knee was used as a basis:
  - No narrowing or *0*+ was defined as normal; when it narrowed by 1/3 or less, it was marked *1*+ and defined as mild; when it narrowed by 2/3 or less but more than 1/3, it was marked *2*+ and defined as moderate; and when it narrowed by more than 2/3, it was marked *3*+ and defined as severe;
  - In some knees, there was no apparent narrowing but only marginal osteophyte was present at medial and/or lateral compartment. In these cases, the severity of marginal osteophyte was first assessed: no marginal osteophyte as *0*+; mild marginal osteophyte as *1*+; moderate marginal osteophyte, *2*+; and severe marginal osteophyte as *3*+. Then, the severity of overall narrowing-marginal osteophyte was defined: i.e., *0*+ narrowing/*2*+ marginal osteophyte was defined as *2*+ moderate narrowing-marginal osteophyte.
3. Accurately to assess all grades and most importantly identify any subtle pathology at TS and compartment, the analysis of knee radiograph under magnification was used as a “mandatory option”.

Second since TS is known as the transverse axis of rotation of the tibia (13-15), osteophyte at TS was defined as Axial OAK-Marker (AOM), an indicator of the pathology of AC over TS and compartment narrowing-marginal osteophyte as Condylar OAK-Marker (COM), an indicator of the pathology of AC and meniscus at compartment and its margin.

Then, AOM and COM can be established as a novel “radiographic OAK-marker” helping diagnose the OAK pathology in kinematical and anatomical aspects (Table 1). Each AOM and COM was previously divided in four grades: *0*+ means normal, *1*+, mild, *2*+, moderate, and *3*+ means severe.

**Table 1:** A novel “radiographic OAK-marker” of AOM and COM in 4-grades to define normal, mild, moderate, and severe OAK

OAK Pathology at site	Osteophyte at Tibial Spine (AOM)	Narrowing-Marginal Osteophyte at Compartment (COM)
OAK Pathology in Severity		

Normal (*0*+)	*0*+	*0*+
Mild (*1*+)	< 1/3	< 1/3
Moderate (*2*+)	> 1/3 but < 2/3	> 1/3 but < 2/3
Severe (*3*+)	> 2/3	> 2/3
**Kellgren & Lawrence grade**	**Kellgren & Lawrence definition**	

Grade 1v ‘Doubtful’	Minute osteophyte, doubtful significance	
Grade 2 ‘Minimal’	Definite osteophyte, unimpaired joint space	
Grade 3 ‘Moderate’	Moderate diminution of joint space	
Grade 4 ‘Severe’	Joint space greatly impaired with sclerosis of subchondral bone	
Grade 4 ‘Severe’	Joint space greatly impaired with sclerosis of subchondral bone	
Grade 4 ‘Severe’	Joint space greatly impaired with sclerosis of subchondral bone	
Grade 4 ‘Severe’	Joint space greatly impaired with sclerosis of subchondral bone	

The Kellgren and Lawrence grading system of radiographic knee OA of the tibiofemoral joint (Kellgren JH, 1963) is presented for comparison purpose.

### Inter-Observer Reliability

In order to assess the accuracy of radiographic OAK-marker in OAK diagnosis, knee radiographs were studied by two surgical fellows (one from orthopedics and another from podiatry) who were specifically trained to read and analyze OAK-marker criteria. Out of 234 knee radiographs, the agreement on the severity of OAK was reached for 211 cases, while on 23 cases, the opinion on the severity differed. It shows 90.2% inter-observer reliability in OAK diagnosis. Different conclusion on diagnosis was related to discrimination between mild and moderate OAK and between moderate and severe OAK. All mild OAK cases were diagnosed in consensus.

### A Cohort of Bilateral Knee Radiographs

A random cohort of both knee radiographs of 1,872 patients who visited orthopedic clinic for knee pain was obtained. These patients had one knee painful and the opposite one non-painful, except for those with severe OAK in which both knees were painful. Out of this set, 1,728 patients’ bilateral knee radiographs (3,456 knees) met the criteria established for radiographic OAK-marker. Then, each knee radiograph was magnified and analyzed based on the criteria of AOM/COM. It showed that the OAK pathology was involved in both knees at almost identical degree. In this analysis, a high or equal value of AOM/COM was interpreted as the severity of given OAK: i.e., *1*+/*0*+ and *1*+/*1*+ as mild OAK, *2*+/*1*+ and *1*+/*2*+, moderate OAK, or *3*+/*2*+ and *3*+/*3*+ as severe OAK and then, recorded in each cell of Table 2. It can be used to define the pathological features of OAK.

**Table 2:** In 3,456 knees radiographs, 198 normal knees (left upper corner); and 3258 OAK based on the grade of AOM/COM

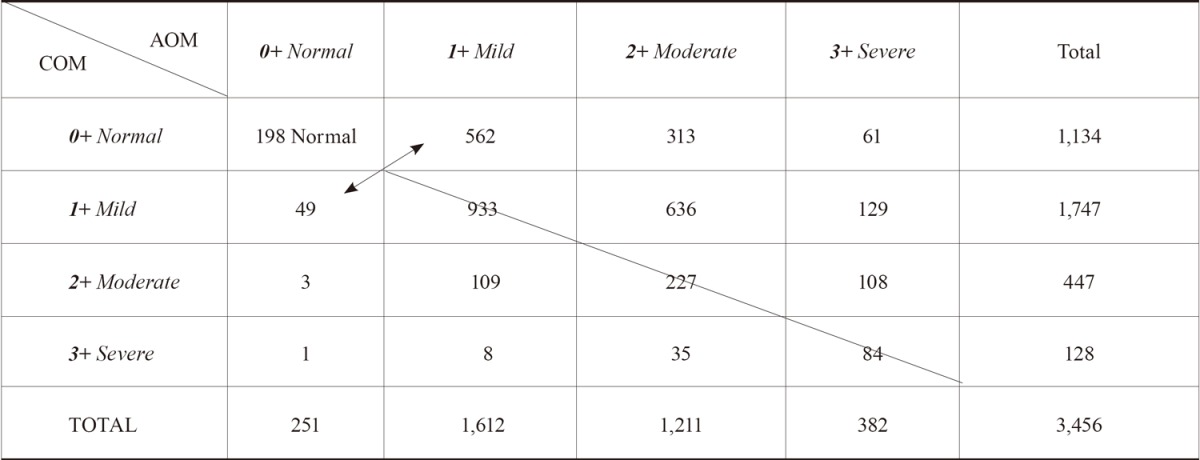

## RESULTS

### Radiographic Pathological Features of OAK

1,728 patients’ both knees radiographs (3,456 knees) were divided in two groups based on the presence or absence of OAK pathology: a) non-OAK group identified by AOM/COM (*0*+/*0*+) as normal knee (left-upper corner of Table 2) constituted 198 knees; and b) OAK group identified by AOM/COM (i.e., *1*+/*0*+, *2*+/*1*+, *3*+/*2*+, etc.) constituted 3,258 knees.

**A) Morphologic Features of Early-Stage Mild OAK.** Among OAK group, 1,544 knees identified by *1*+/*0*+, *0*+/*1*+, and *1*+/*1*+ were defined as mild OAK.

**Figure 3:**
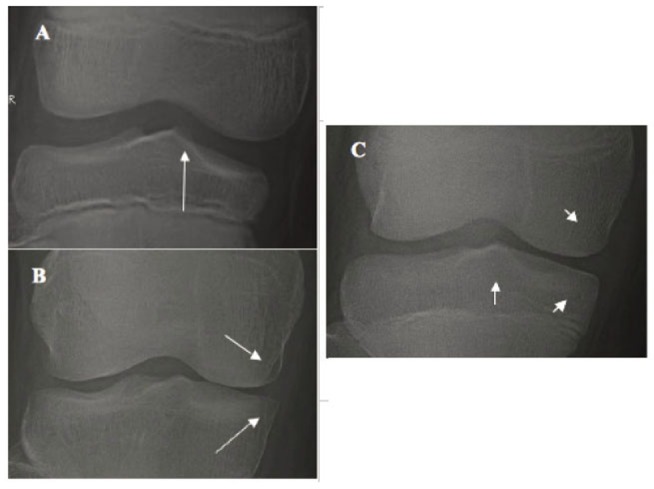
Earliest change of mild OAK. A, An arrow indicates a subtle osteophyte at MTS without narrowing; B, An arrow indicates mild medial compartment narrowing with a beaky tibial articular margin and squaring of femoral articular margin without osteophyte at TS; and C - Normal knee: A short-vertical arrow indicates a smooth, round MTS without osteophyte; and two-short slant arrows indicate a smooth, round medial compartment margin without narrowing.

Among 1,544 mild OAK, some knees showed a subtle change at TS without any change at compartment and some knees showed a subtle change at compartment without any change at TS (a double–headed arrow in Table 2). These subtle changes were considered as early-stage of mild OAK. The morphologic features of early-stage mild OAK under magnification were the following:

- A minute osteophyte at TS (Fig. 3A) *vs.* smooth, round TS in normal knee (Fig. 3C); and
- Suspicious narrowing or flattening, or beaky, squaring of tibial or femoral compartment margin (Fig. 3B) *vs.* equal spaces between medial and lateral compartments and smooth, round peripheral margin in normal knee (Fig. 3C).

According to the definition of OAK pathology in current textbooks (14, 15), these subtle, suspicious changes are considered as “definitive” early-stage mild OAK pathology:

- A minute osteophyte at TS indicates that mild hypertrophic ossification of AC lined over TS has already occurred as “attempted repair”;
- Suspicious compartment narrowing or flattening indicates that very mild thinning of AC and meniscus at compartment has occurred as a “progressive loss”; and
- Beaky, squaring of tibial or femoral compartment margin indicates that mild hypertrophic ossification of AC at the compartmental margins has already occurred as “attempted repair”.

This analysis demonstrates morphologic features of early-stage mild OAK pathology.

**Figure 4:**
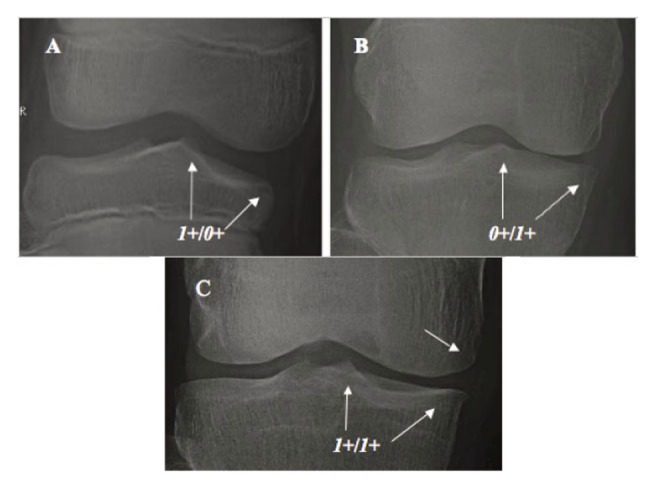
Mild OAK. A, TS-dominant group: *1*+/*0*+, mild osteophyte at TS without narrowing; B, Compartment-dominant group: *0*+/*1*+, mild medial narrowing with a beaky tibial marginal change without osteophyte at TS; and C -Co-dominant groups: *1*+/*1*+. An arrow at medial femoral condyle indicates squaring of femoral articular margin.

**B) The Initial Site of OAK Development and Stage of Mild OAK.** The initial site where OAK to start remains unknown. To address this important question, 1,544 cases of mild OAK were analyzed. 562 knees (*1*+/*0*) with a minute osteophyte at TS without narrowing (Fig. 4A) and 49 knees (*0*+/*1*) with suspicious narrowing-marginal osteophyte without osteophyte at TS (Fig. 4B) were already defined in previous section as “early-stage” mild OAK. Since the value (severity) of *1*+/*0*+ and *0*+/*1*+ is smaller (milder) than that of *1*+/*1*+, 933 knees (*1*+/*1*+) with both osteophyte at TS and narrowing-marginal osteophyte (Fig. 4C) were considered as “late-stage” mild OAK. This analysis suggested two important facts that: a) among 611 early-stages mild OAK, 562 or 92.0% started at TS; and 49 or 8.0% at compartment. Since TS is known as the axis of the tibia, it is kinematically logical that OAK begins sooner at TS than at compartment; and b) mild OAK can be classified as early and late stages.

This analysis helps discover the initial site of OAK development: 92.0% of OAK starts at TS and 8.0% of OAK at compartment; and classify mild OAK as early and late stages.

### New Radiographic Classification System of OAK

The emergence of information on the initial OAK development site and early and late stage of mild OAK prompted to consider the site and stage-specific radiographic classification system of OAK to help classify OAK in severity and stage based on the anatomical site.

First for the anatomical site, OAK cases are classified in three groups: *TS-dominant* group; *compartment-dominant* one; and *co-dominant*-group:

- *TS-dominant* group*:* the value of AOM is greater than that of COM: i.e., *1*+/*0*+ in mild OAK, *2*+/*1*+ in moderate OAK, and *3*+/*1*+ in severe OAK;
- *Compartment-dominant* group: the value of COM is greater than that of AOM: i.e., *0*+/*1*+ in mild OAK, *1*+/*2*+ in moderate OAK, and *1*+/*3*+ in severe OAK; and
- *Co-dominant* group: the value of AOM and COM is equal: i.e., *1*+/*1*+ in mild OAK, *2*+/*2*+ in moderate OAK, and *3*+/*3*+ in severe OAK.

Second for the stage in severity, the method that mild OAK identified by *1*+/*0*+ and *0*+/*1*+ defined as early-stage and *1*+/*1*+ as late-stage is used to define the stage of moderate and severe OAK:

- The knees identified by *2*+/*0*+ and *0*+/*2*+, and *3*+/*0*+ and *0*+/*3*+ are defined as early-stage of moderate and severe OAK; and those identified by *2*+/*2*+ and *3*+/*3*+ as late-stage respectively; and
- Those between the early and late-stages identified by *2*+/*1*+ and *1*+/*2*+ and those identified by *3*+/*1*+ and *3*+/*2*+, and *1*+/*3*+ and *2*+/*3*+ are defined as “intermediate-stage” of moderate and severe OAK respectively.

**Figure 5:**
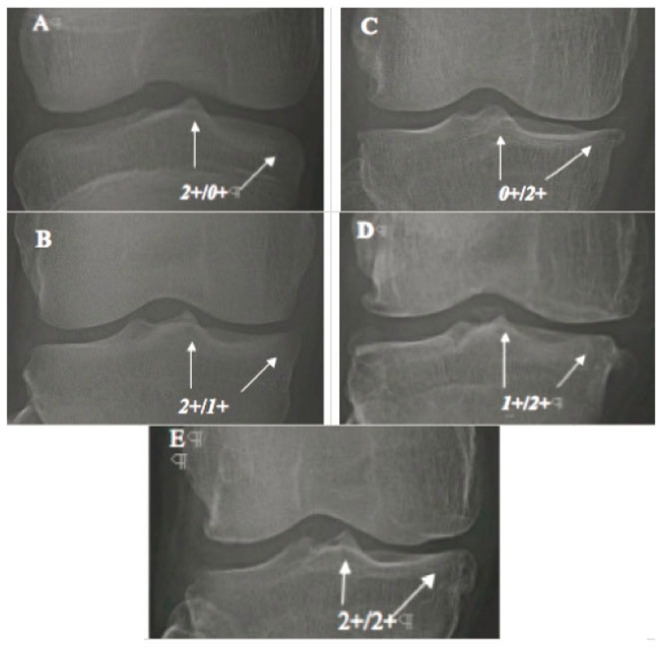
Moderate OAK. A,B - TS-dominant group: A: *2*+/*0*+; B: *2*+/*1*+; C,D - Compartment dominant group: C: *0*+/*2*+; D: *1*+/*2*+; and E - Co-dominant groups, *2*+/*2*+. Note that: in figure C (*0*+/*2*+), mild to moderate narrowing with moderate tibial marginal osteophyte; and in figure D (*1*+/*2*+), moderate narrowing with moderate tibial marginal osteophyte.

**Figure 6:**
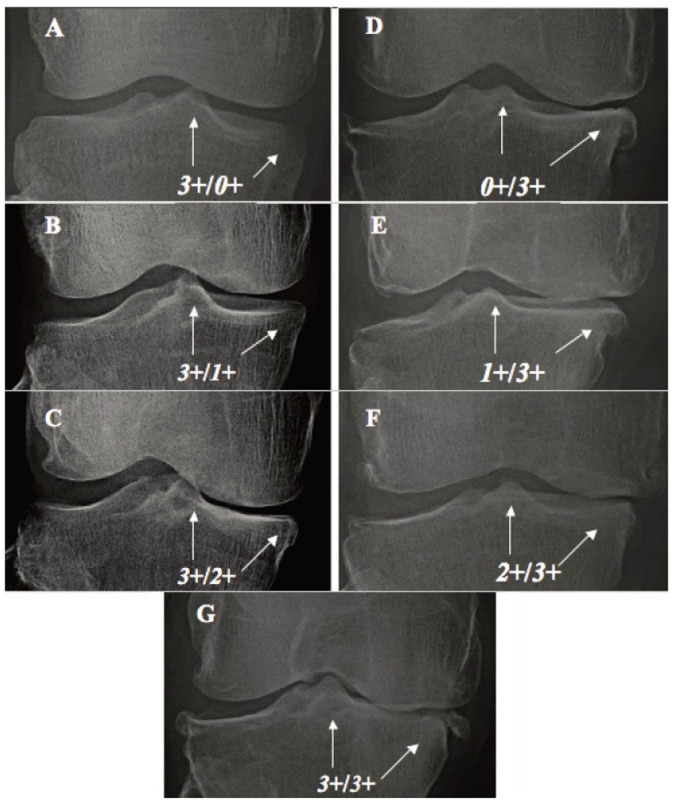
Severe OAK. A,B,C - TS-dominant group: A: *3*+*0*+; B: *3*+/*1*+; C: *3*+/*2*+; D,F,F - Compartment dominant group: D: *0*+/*3*+; E: *1*+/*3*+; F: *2*+/*3*+; and G - Co-dominant groups, *3*+/*3*+.

Then, all OAK cases can be classified based on the anatomical site. Their severity and stage can be expressed as grades of AOM/COM (Table 3):

1. Mild OAK includes 562 cases (*1*+/*0*+)of early-stage (Fig. 4A) of *TS-dominant* group and 49 cases (*0*+/*1*+) of early-stage (Fig. 4B) of *compartment-dominant* group. Late-stage of Mild OAK includes 933 knees with *1*+/*1*+ (Fig. 4C) of *co-dominant* group;
2. Moderate OAK includes 313 cases (*2*+/*0*+) of early-stage (Fig. 5A) and 636 cases (*2*+/*1*+) of intermediate-stage (Fig. 5B) of *TS-dominant* group and three cases (*0*+/*2*+) of early-stage (Fig. 5C) and 109 cases (*1*+/*2*+) ofintermediate-stage (Fig. 5D) of *compartment-dominant* group. Late-stage of moderate OAK includes 227 cases with *2*+/*2*+ (Fig. 5E) of *co-dominant* group; and
3. Severe OAK includes 61 cases (*3*+/*0*+) of early-stage (Fig. 6A) and 129 cases with *3*+/*1*+ (Fig. 6B) and 108 cases with *3*+/*2*+ (Fig. 6C) of intermediate-stage of *TS-dominant *group and one case (*0*+/*3*+) of early-stage (Fig. 6D), eight cases with *1*+/*3*+ (Fig. 6E) and 35 cases with *2*+/*3*+ (Fig. 6F) of intermediate-stage of *compartment-dominant *group. Late-stage of severe OAK includes 84 cases with *3*+/*3*+ (Fig. 6G) of *co-dominant* group.

**Table 3:** Radiographic Classification System of OAK to classify and diagnose OAK in severity and stage based on the anatomical site as TS-dominant group, compartment-dominant group, and co-dominant group

Anatomical Site		*Tibial Spine-Dominant* Group (1,809 knees in 55.5%)	*Compartment-Dominant* Group (205 knees in 6.3%)	*Co-Dominant*Group (1,244 knees in 38.2%)
Severity & Stage				

Mild OAK	Early Stage	*1*+/*0*+ (562)	*0*+/*1*+ (49)	
	Late Stage			*1*+/*1*+ (933)
Moderate OAK	Early Stage	*2*+/*0*+ (313)	*0*+/*2*+ (3)	
	Intermediate Stage	*2*+/*1*+ (636)	*1*+/*2*+ (109)	
	Late Stage			*2*+/*2*+ (227)
Severe OAK	Early Stage	*3*+/*0*+ (61)	*0*+/*3*+ (1)	
	Intermediate Stage	*3*+/*1*+ (129)	*1*+/*3*+ (8)	
		*3*+/*2*+ (108)	*2*+/*3*+ (35)	
	Late Stage			*3*+/*3*+ (84)

This classification system shows that *TS-dominant* group (1,809 knees) outnumbers *compartment-dominant* one (205 knees) at about 9:1 ratio.

With this classification system, every OAK can accurately be diagnosed in severity and at stage.

### Application of New Classification System to Improve OAK Diagnosis

First this classification system based on AOM/COM grading and using “magnification” of knee x-rays significantly decreases the number of missed early cases of OAK (Fig. 4): a) 611 knees [562 knees (*1*+/*0*+) + 49 knees (*0*+/*1*+)], which constitute 18.8% of all OAK cases, were diagnosed as early-stage mild OAK; b) 933 knees (*1*+/*1*+), which constitute 28.6% of all OAK cases, were diagnosed as late-stage mild OAK.

However, K&L system without ‘magnification” might have diagnosed only 49 or 8% of early-stage of compartment-dominant mild OAK (*0*+/*1*+) and failed to diagnose 562 or 92% of early-stage of TS-dominant mild OAK (*1*+/*0*+).

Second upon surveying a number of recent studies (3, 5), new classification system with four-grading of osteophyte at TS has eliminated a difficulty in diagnosing early-stage of moderate and severe OAK of *TS-dominant* group presented by K&L system (top row in Table 2):

- Diagnosed 313 OAK with moderate osteophyte at TS without narrowing (*2*+/*0*+) as early-stage moderate OAK;
- 61 OAK with severe osteophyte at TS without narrowing (*3*+/*0*+) as early-stage severe OAK.

However, K-L system, which describes osteophyte at TS without detailed-grading, might have experienced a difficulty in grading those 374 early-stages moderate OAK (*2*+/*0*+) and severe OAK (*3*+/*0*+), which constitute 11.5% of all OAK cases, or might have missed them as *0* grade.

Third, new classification system has also helped in preventing K-L system from underdiagnosing intermediate stages of moderate and severe OAK of *TS-dominant* group (the right to a diagonal line in Table 2):

- Diagnosed 636 OAK with moderate osteophyte at TS with mild narrowing (*2*+/*1*+) as intermediate-stage moderate OAK;
- 129 OAK with severe osteophyte at TS with mild narrowing (*3*+/*1*+) as intermediate-stage severe OAK; and
- 108 OAK with severe osteophyte at TS with moderate narrowing (*3*+/*2*+) as intermediate-stage severe OAK.

However, K&L system could have underdiagnosed these 873 OAK with *2*+/*1*+, *3*+/*1*+, and *3*+/*2*+, which are 26.8% of all OAK cases, as mild OAK, mild OAK, and moderate OAK respectively.

This analysis shows that new classification system has eliminated impact of inexact description of K&L system that might have missed at least 562, or 92.0% early-stage mild OAK or perhaps entire 1,544 mild OAK (47.4% of all OAK cases). All missed OAK may deteriorate to be a contributing factor in an increasing incidence of OAK.

## DISCUSSION

The data presented above demand for the explanation of one issue. Difficulties in considering osteophyte at TS only as radiographic evidence of OAK as in K&L system rather than the criteria for radiographic grading of OAK may relate in part to the “space” between TS and distal femoral condyle as seen on knee radiographs. It gives a false impression that there is no contact between them. Then, osteophyte at TS may be viewed as no more than an irrelevant bump on tibial plateau. However, this space is filled with radiolucent AC lined over TS and inner border of articular surface of distal femoral condyle. This radiolucent AC minimizes friction between those surfaces. The same can be said about the space between articular surfaces of femoral and tibial peripheral margins, which is filled with AC and meniscus, and it is meniscus to shock-absorb protecting AC from the load at gait. It shows that contact between articular surfaces of TS and distal femoral condyle is AC-AC “direct” contact, while contact between articular surfaces of femoral and tibial peripheral margins is AC-meniscus-AC “indirect” contact. It suggests that shock absorption capability would be lower at AC over TS than at AC at compartment margin upon carrying the load at gait. Moreover TS is known as an axis of rotation of the tibia. Then, from tribological and kinematical aspects, it is understandable why OAK begins at TS sooner than at compartment and its margin at 9:1 ratio.

Then, how AC at these two sites will respond to the overload (overweight or obesity) at gait, which is a well-known mechanical risk factor of OAK pathology (18, 19)? An answer to this question can be found from the morpho-pathology of osteophyte as it seen on radiographs (Fig. 1):

- AC at TS elicits vertically-oriented hypertrophic osteophyte as an attempt repair for damaged AC; and
- AC at compartment margins elicits horizontally-oriented-hypertrophic osteophyte as such.

This discussion provides tribological, kinematical, and morpho-pathological evidence that a subtle osteophyte at TS is the first and foremost radiographic sign of OAK.

## CONCLUSION

An analysis of radiographs of OAK allowed for the development of radiographic OAK-marker. It helped in identifying the initial site of OAK. It allowed for accurately diagnosing early stages OAK without obtaining any additional test such as MRI and establishing a new radiographic classification system to diagnose all OAK in severity and stages. Thus, this system compensates in part for shortcoming of Kellgren-Lawrence classification system. All these would make a difference in the future patients’ care. It also allowed for defining the pathologic features of OAK and suggests two recommendations:

The pathologic features include:

1. TS is more common initial OAK development site than compartment: and
2. the OAK pathology occurs more commonly at TS than at compartment.

The recommendations include:

1. The knee with a minute osteophyte at TS and/or narrowing-marginal osteophyte may be considered as OAK; and
2. new radiographic classification system can be a useful tool for accurate diagnosis and epidemiological assessment of OAK.

## References

[1] Kellgren JH, Lawrence JS (1957). Radiographic Assessment of Osteoarthritis. Ann. Rheumatologic Disease.

[2] Kellgren JH, Jeffrey M, Ball J (1963). The epidemiology of chronic rheumatism. Atlas of standard radiographs.

[3] Spector TD, Cooper C, Cushnagham J (1992). A Radiographic atlas of knee osteoarthritis.

[4] Spector TD, Hart DJ, Byrne J, Harris PA (1993). Definition of osteoarthritis of the knee for epidemiological studies. Ann. Rheum. Dis.

[5] Hart DJ, Spector TD (1995). The classification and assessment of osteoarthritis. Baillieres Clin. Rheumatol.

[6] Schiphof D, Boers M, Bierma-Zeinstra SMA (2008). Differences in descriptions of Kellgren and Lawrence grades of knee osteoarthritis. Ann. Rheum. Dis.

[7] Schiphof D, de Klerk BM, Kerkhof HJM, Hofman A (2011). Impact of different descriptions of the Kellgren and Lawrence classification criteria on the diagnosis of knee osteoarthritis. Ann. Rheum. Dis.

[8] (1998). Gray’s Anatomy for the Human Body.

[9] Giorgi B (1956). Morphologic variation of the intercondylar eminence of the knee. Clin. Orthop.

[10] Girgis FG, Marshall JL, Monajem A (1975). The Cruciate ligaments of the knee joint. Anatomical, functional, and experimental analysis. Clin. Orthop. Relat. Res.

[11] Mankin HJ, Grodzinski AJ, Buckwalter JA (2008). AAOS: Articular cartilage and Osteoarthritis: Orthopedic Basic Science.

[12] Simon Sheldon R (1994). AAOS: Orthopedic Basic Science.

[13] Brantigan OC, Voshell AF (1941). The Mechanism of the Ligament and Menisci of the Knee Joint. JBJS Am.

[14] Shaw JS, Eng M, Murray DG (1974). The Longitudinal Axis of the Knee and the Role of the Cruciate Ligaments in Controlling Transverse Rotation. JBJS Am.

[15] Trent Peter S, Walker Peter S, Wolf Barry (1976). Ligament Length Pattern, Strength and Rotational Axis of the Knee Joint. Clin. Ortho.

[16] Apostolos H. Karantanas (2007). Magnetic Resonance Imaging Findings in Early Osteoarthritis of the Knee, © Touch briefings, 2007. European Musculo-Skeletal Review.

[17] Kurtz SM, Lau Edmund, Ong Kevin, MA Ke Zhao (2009). Future Young Patient Demand for Primary and Revision Joint Replacement. Clinical Orthopedics and Related Research.

[18] Radin EL, Paul IL, Rose RM (1972). Role of mechanical factors in pathogenesis of primary osteoarthritis. Lancet.

[19] Kohatsu ND, Schurman DJ (1990). Risk Factors for the Development of Osteoarthritis of the Knee. Clinical Orthopedics and related research.

